# Using Machine Learning Algorithms to Predict Hospital Acquired Thrombocytopenia after Operation in the Intensive Care Unit: A Retrospective Cohort Study

**DOI:** 10.3390/diagnostics11091614

**Published:** 2021-09-03

**Authors:** Yisong Cheng, Chaoyue Chen, Jie Yang, Hao Yang, Min Fu, Xi Zhong, Bo Wang, Min He, Zhi Hu, Zhongwei Zhang, Xiaodong Jin, Yan Kang, Qin Wu

**Affiliations:** 1 Department of Critical Care Medicine, West China Hospital, Sichuan University, Chengdu 610041, China; yisongcheng01@163.com (Y.C.); yangjie@wchscu.cn (J.Y.); nonamedoctor@163.com (H.Y.); carrie_613@163.com (M.F.); zhongxivip2006@163.com (X.Z.); wchicu@126.com (B.W.); hemin19910306@wchscu.cn (M.H.); huzhi1111@hotmail.com (Z.H.); zhangzhongweihxyy@163.com (Z.Z.); zh_jxd@163.com (X.J.); kangyan@scu.edu.cn (Y.K.); 2 Department of Neurosurgery, West China Hospital, Sichuan University, Chengdu 610041, China; chaoyuechen01@gmail.com

**Keywords:** hospital acquired thrombocytopenia, machine learning, predictive models, surgery, critical care

## Abstract

Hospital acquired thrombocytopenia (HAT) is a common hematological complication after surgery. This research aimed to develop and compare the performance of seven machine learning (ML) algorithms for predicting patients that are at risk of HAT after surgery. We conducted a retrospective cohort study which enrolled adult patients transferred to the intensive care unit (ICU) after surgery in West China Hospital of Sichuan University from January 2016 to December 2018. All subjects were randomly divided into a derivation set (70%) and test set (30%). ten-fold cross-validation was used to estimate the hyperparameters of ML algorithms during the training process in the derivation set. After ML models were developed, the sensitivity, specificity, area under the curve (AUC), and net benefit (decision analysis curve, DCA) were calculated to evaluate the performances of ML models in the test set. A total of 10,369 patients were included and in 1354 (13.1%) HAT occurred. The AUC of all seven ML models exceeded 0.7, the two highest were Gradient Boosting (GB) (0.834, 0.814–0.853, *p* < 0.001) and Random Forest (RF) (0.828, 0.807–0.848, *p* < 0.001). There was no difference between GB and RF (0.834 vs. 0.828, *p* = 0.293); however, these two were better than the remaining five models (*p* < 0.001). The DCA revealed that all ML models had high net benefits with a threshold probability approximately less than 0.6. In conclusion, we found that ML models constructed by multiple preoperative variables can predict HAT in patients transferred to ICU after surgery, which can improve risk stratification and guide management in clinical practice.

## 1. Introduction

Platelets are directly involved in thrombus formation and inflammatory regulation, and thrombocytopenia is a common complication in intensively ill patients [1]. The incidence of hospital acquired thrombocytopenia (HAT) in adult critically ill patients admitted to the intensive care unit (ICU) ranges from 8.3% to 67.6%, and the incidence of HAT during ICU treatment can reach 14~44% [2,3]. The current evidence reveals that HAT is associated with increased bleeding and transfusion risk, ICU mortality and length of stay, and need for organ support [3,4].

HAT is a common phenomenon after major operations such as hip replacement, abdominal surgery, and heart surgery. Because of tissue damage and blood loss, the platelet count usually drops to the lowest point between 1 and 4 days after surgery, rises back to preoperative levels between 5 and 7 days, and reaches the highest level around the 14th day [5]. It seems to be a short, transient, and reversible clinical process, which is not related to the patient’s postoperative recovery. However, more and more evidence show that this is not a meaningless process. A study reported that platelets < 75 × 10^9^/L after cardiac surgery is an independent risk factor for adverse events such as acute kidney injury (AKI), infection, and stroke [6]. Tew et al. [7] found that the platelet count of children undergoing cardiac surgery was negatively correlated with serum creatinine, and the lowest platelet count was closely related to the severity of AKI. Therefore, identifying patients at risk of developing HAT transferred to ICU after surgery is important for risk stratification, improving quality of care, and facilitating clinical decision-making.

There are several risk scores proposed to predict the likelihood of heparin-induced thrombocytopenia (HIT), the Warkentin 4T score was common used in practice [8] and the HIT expert probability score showed a very good negative predictive value (NPV, 97%) for ruling out HIT [9]. A systematic review found that a PLASMIC score (contains seven variables) threshold of over or equal to five is associated with high sensitivity and NPV of predicting thrombotic thrombocytopenic purpura (TTP) in patients with suspected TTP [10]. Another study found that acquired thrombocytopenia after transcatheter aortic valve replacement was strongly associated with baseline (low platelet count, leucocyte count), procedural (eg. major vascular complication), and post-procedural adverse events (sepsis, AKI); however, they did not develop a predictive model that can be used for predicting acquired thrombocytopenia [11]. Thus, to the best of our knowledge, there is currently no study that has established diagnostic models by machine learning (ML) method to evaluate the occurrence of HAT in patients after surgery. With the rapid development of ML technology, it has been widely used in various diseases [12,13,14,15]. The advantage of ML algorithms is that they can explain high-order nonlinear interactions of predictors and obtain more stable predictions [16]. In this study, we aimed to use ML algorithms with the clinical and laboratory test data before surgery to predict the occurrence of HAT in patients transferred to ICU after surgery.

## 2. Methods

### 2.1. Study Design

This study used a database of patients who transferred to ICU after surgery in West China Hospital of Sichuan University. This single-center database retrospectively enrolled the adult patients (≥18 years old) transferred to ICU after surgery between January 2016 and December 2018. The exclusion criteria were: (1) thrombocytopenia before surgery (platelets < 100 × 10^9^/L); (2) age < 18 years; (3) taking thrombocytopenia drugs within 3 months; (4) history of acute blood loss or transfusion during the perioperative period; (5) primary diseases that cause thrombocytopenia such as aplastic anemia, hematological malignancies, etc.

### 2.2. Data Collecting and Predictors

First, we obtained demographic characteristic, vital sign, comorbidity (hypertension and diabetes), Acute Physiology and Chronic Health Evaluation II (APACHE II), sequential organ failure assessment (SOFA), and laboratory indicators from the database. Laboratory indicators were measured after admission and, whenever necessary, according to attending physicians’ judgment. The laboratory value was determined in the Laboratory Department of West China Hospital within 2 h after the blood was collected. SOFA and APACHE II were evaluated by the attending physician who saw the transferred patients.

White blood cell count (WBC), hemoglobin, and platelets were analyzed by an automated hematology analysis system, Beckman Coulter LH750 (Beckman Coulter Inc., Brea, CA, USA). Activated partial thromboplastin time (APTT), prothrombin time (PT), fibrin, and fibrinogen degradation products (FDP) were measured by a Sysmex CA-7000 analyzer (Siemens Healthcare Diagnostics, Shanghai, China). Procalcitonin and interleukin-6 were tested by a Cobas S6000 Hitachi (Roche Diagnostics, Quebec, H7V 4A2, Canada).

### 2.3. Endpoint

Since the normal range of platelets in the Chinese population is lower than that of the European and American population [1,17], according to expert consensus of Critical Care Medicine Committee of Chinese Medical Association, HAT was defined as platelets < 100 × 10^9^/L that transferred to ICU after surgery in this study [18].

### 2.4. Machine Learning

To achieve the purpose of the research, 7 supervised ML algorithms were used to develop classification models: (1) Random Forest (RF), (2) Gradient Boosting (GB), (3) Logistic Regression (LR), (4) XGBoost, (5) multi-layer perceptron (MLP), (6) support vector machine (SVM), and (7) K-nearest neighbor (KNN), since they are commonly used and can identify non-linear relationships between variables [19,20]. First, we chose the algorithm of the model and some model parameters arbitrarily, and provided derivation data for each model. Together with the training step, the model gradually adjusts some trainable parameters to optimize performance by itself. After training, all model parameters were fixed.

RF builds a Bagging ensemble based on decision tree learner, and further introduces random attribute selection in the training process of decision tree. It builds each tree using random features of random variables, then finally returns the average predictions of each tree [21]. GB is a technique that learns from its mistakes, and it iterates multiple regression trees to make joint decisions. When using the squared error loss function, each regression tree will learn the conclusions and residuals of all previous trees and fit them to obtain the current residual regression tree [16]. LR (aka logit, MaxEnt) is an easy-to-implement and excellent performance classification model for linear separable problems, it implements regularized logistic regression using the “liblinear” [22]. XGBoost is a novel boosting tree-based ensemble algorithm and has been widely used due to its ability of employing both continuous and categorical variables, interpretably, without the need for scaling, and its capacity for handling of sparsity [23]. XGBoost improves the classification accuracy iteratively by optimizing a custom objective function (an instance of process, also called “boosting”). MLP is a feedforward artificial neural network model with multiple neuron layers. MLP is implemented using many parameters, so that they can flexibly approximate any smooth function. Except for the last layer with sigmoid activation function for binary outcome, all layers have a ReLu activation function [24]. SVM constructs hyperplanes of the covariates’ space that separates the observations according to their category. The separation is achieved by using kernel functions to expand the feature space to allow non-linear relationships between results and covariates so that complex relationships can be detected and modeled [25,26]. KNN is a data mining algorithm based on statistics. For newly input test samples, it selects k nearest neighbor samples with the smallest Euclidean distance from the test sample in the training data set, and makes predictions based on the information of these k nearest neighbor samples [27].

### 2.5. Derivation and Test Set and Cross-Validation

The derivation-test set is an effective strategy to reduce the model overfitting. In this study, all subjects were randomly divided into derivation set and test set at a ratio of 7:3. The models were trained in the derivation set and the test set was not used until the models were constructed.

Furthermore, a k-fold cross-validation was proposed to better estimate the performance of the model and has been used in various literatures [28,29,30]. Briefly, data are divided into k subsets of similar size, the model can be trained on every subset but 1 and then tested on that left-out subset, so that k times of training and testing of the model can be completed, and finally the mean value of k test results is returned.

During the training of models, hypermeters of models were optimized with a grid search algorithm. Grid research is a method of optimizing hypermeters through exhaustive search (Supplemental Table S1). In this study, 10-fold cross-validation was performed in the derivation set to select the optimal parameters of the models by evaluating their performances.

### 2.6. Feature Selection and Oversampling

To improve the interpretability and generalization ability of models, we performed feature selection to keep only relevant variables in the construction of models using the filter, wrapper, or embedding method in different algorithms. For example, in the Boruta algorithm [31], a wrapper method built based on random the forest algorithm was used to calculate the importance of features.

Since the samples of HAT and non-HAT patients is unbalanced, we used the Synthetic Minority Oversampling Technique (SMOTE) to compensate for unbalanced data. The SMOTE is an oversampling algorithm that analyzes minority samples and artificially synthesize new samples into the data set, it is an improved method to reduce overfitting of models based on random oversampling [32].

### 2.7. Statistical Analysis

Data were presented as mean and standard deviation or median and interquartile ranges (IQR) according to the distribution of continuous variables, and the differences were compared by a *t*-test or Mann–Whitney U-test. Categorical variables were presented as numbers and percentages, and examined by the chi-square test.

We describe algorithm performance in the test set by the area under the curve (AUC) to quantify how well the machine learning models discriminated between those who were with and without HAT. Additionally, other performance indicators such as sensitivity, positive predict value (PPV), specificity, and negative predict value (NPV) were also calculated for measurement of each model [33,34]. To further explore which model has advantages among these 7 ML models and whether they were worth using in clinical practice, decision curve analysis (DCA) was performed to evaluate the models [35]. All ML models were developed in Python 3.7 (Python Software Foundation, Fredericksburg, VA, USA). A two-sided *p*-value of <0.05 was considered statistically significant.

## 3. Results

### 3.1. Study Population

**Figure 1:**
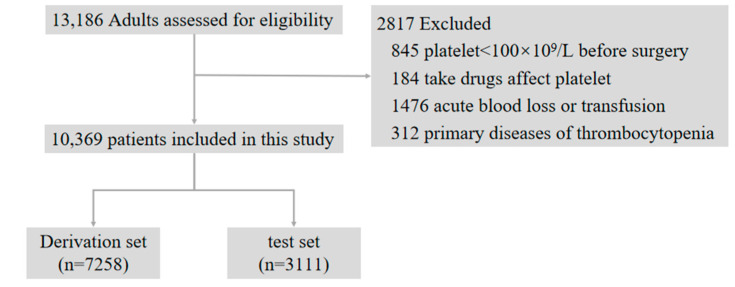
Flow chart of the patients included in this study.

Figure 1 shows the patient flow chart, 2817 patients were excluded according to the exclusion criteria, and a total of 10,369 patients enrolled in this study, with a mean age of 54.4 ± 15.2 years, and 6117 (59.0%) male. The derivation set comprised 7258 patients, in 954 (13.1%) of which HAT occurred during ICU stays, and the test set comprised 3111 patients, in 400 (12.9%) of which HAT occurred. Basically, there were no statistical differences in clinical characteristics and laboratory indicators between the derivation and test set (Supplemental Table S2).

Patients’ baseline characteristics are listed in Table 1. In general, patients in whichHAT occurred were older, and had a higher rate of hypertension and diabetes. In the HAT group, the platelet, platelet crit, hemoglobin, and albumin level were significantly lower than the non-HAT group. On the other hand, the coagulation predictors (activated partial thromboplastin time, prothrombin time, thrombin time, and fibrinogen degradation products) were significantly higher and the hospital stays were longer than those without HAT (*p* < 0.001).

### 3.2. HAT and Adverse Outcomes

**Figure 2:**
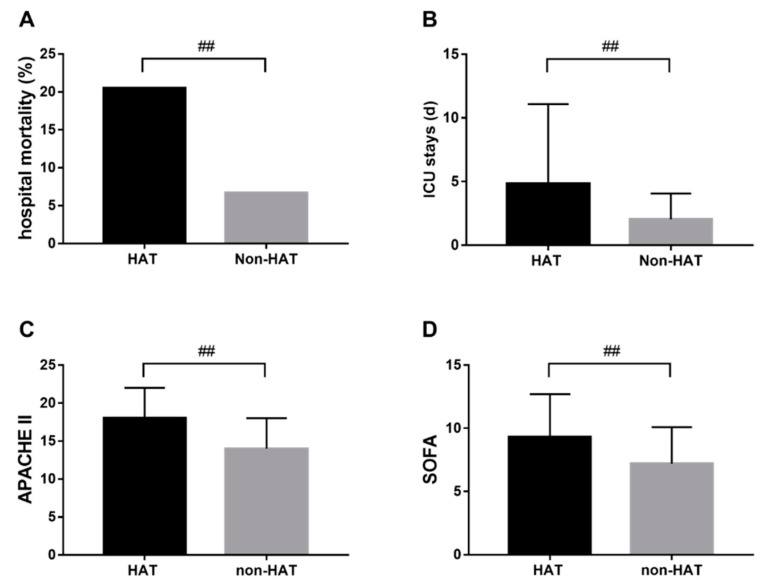
Hospital mortality (**A**), ICU stays (**B**), APACHE II (**C**), and SOFA (**D**) in HAT and non-HAT patients. ^##^: *p* < 0.001.

Generally, patients with HAT during ICU hospitalization were more likely to have adverse outcomes. Separately, 278 patients (20.5%) died in the HAT group (Figure 2A), and the mortality was approximately three times that of non-HAT patients (6.7%). The average length of ICU stay was 4.8 days in patients with HAT, longer than 2.0 days for non-HAT patients (Figure 2B). Similarly, the SOFA and APACHE II score of HAT patients was much higher than non-HAT patients (Figure 2C,D).

### 3.3. Performance of ML Models

**Figure 3:**
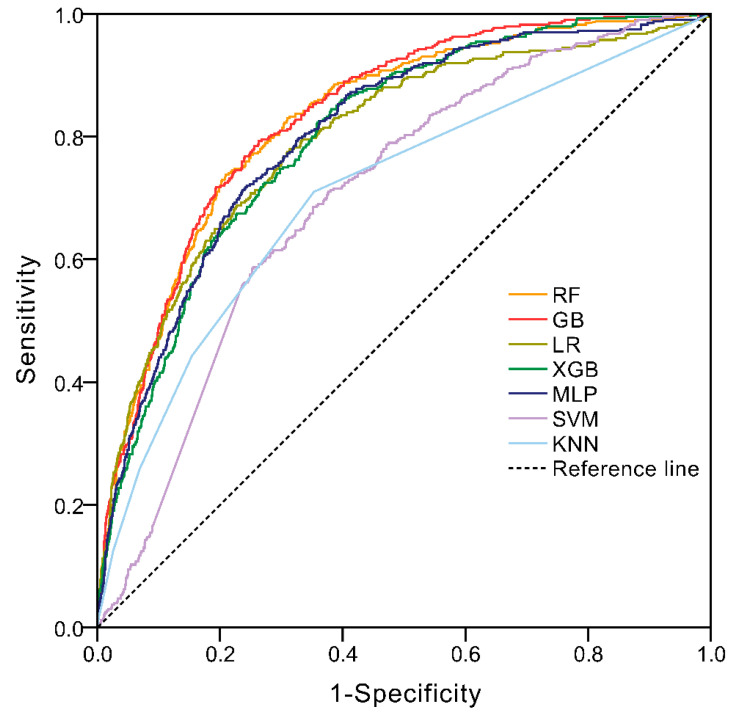
ROC of machine learning models in predicting HAT in the test set.

Figure 3 showed that the ML models had variable discriminability in predicting the occurrence of HAT. Table 2 listed the performance of each model, the AUC of all seven ML models exceeded 0.7, and the highest was GB (AUC = 0.834, 95% CI: 0.814–0.853, *p* < 0.001), with a sensitivity of 79.3% and specificity of 73.7%. The highest sensitivity achieved by XGB was 84.5%, with a specificity of 61.9%. The best specificity was Random Forest (79.1%) with a sensitivity of 73.8%. The highest PPV was RF (34.3) and the highest NPV was SVM (99.1). The AUC of ML models before feature selection were shown in Supplementary Table S3 and Figure S1.

**Figure 4:**
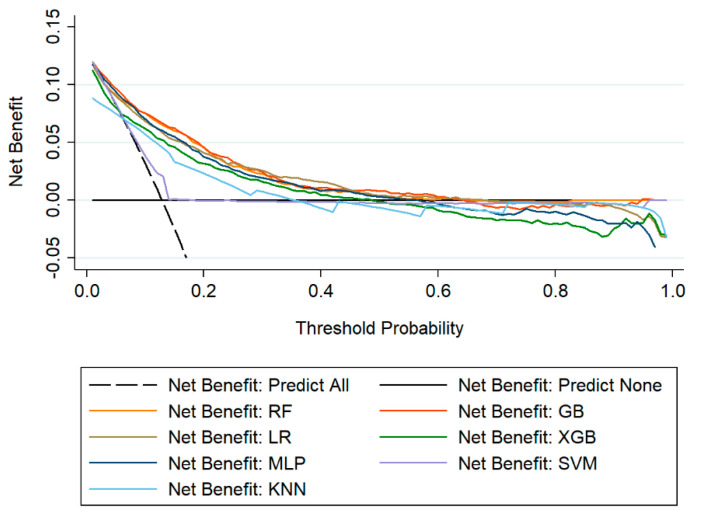
Decision curve analysis of machine learning models for prediction of HAT in the test set.

Likewise, the DCA (Figure 4) demonstrated that the net benefit of all ML models surpassed that of predicting all or none patients having HAT when threshold probability was approximately less than 0.6.

### 3.4. The Comparison of Machine Learning Models

The two highest AUCs of ML models were GB (0.834, 0.814–0.853, *p* < 0.001) and RF (0.828, 0.807–0.848, *p* < 0.001). There was no difference between GB and RF (0.834 vs. 0.828, *p* = 0.293), however, these two were better than the remaining five models (*p* < 0.001) (Figure 3 and Table 2). Consistently, DCA showed that the net benefit of GB and RF were similarly and slightly higher than other models (Figure 4).

### 3.5. Important Features of ML Models

To gain insights into the relevance of feature, after calculating the importance of each feature, the five most important features are shown in Table 3. Platelet, procalcitonin, and prothrombin time seemed to be the important features in three ML models, and activated partial thromboplastin time, direct bilirubin, and interleukin-6 appeared in two models.

## 4. Discussion

In this large retrospective cohort study of over 10,000 patients transferred to ICU after surgery, we developed and compared seven supervised ML algorithms in predicting the occurrence of HAT in the studied population. The GB and RF were both found to have the best performance, including improved AUCs and net benefits. To the best of our knowledge, this is the first study that comprehensively examined the efficacy of ML models for predicting HAT in a large population of adult patients transferred to ICU after surgery.

Platelets are small pieces of cytoplasm that detached from the cytoplasm of mature megakaryocytes in the bone marrow. Healthy human bone marrow megakaryocytes produce about 150 × 10^6^ platelets every day, and their lifespan is about 10 days [36]. Platelets play an important role in primary hemostasis, tissue repair, inflammation regulation, and immune responses [37]. Under pathological conditions, platelets may promote excessive inflammation and are associated with organ damage such as AKI and acute lung injury. In this report, the occurrence of HAT was 13.1%, this is a little higher than previously reported in the literature (5–10%); however, it is worth mentioning that their HAT was defined as less than 150 × 10^9^/L [38,39]. The type of surgery affects the rate of platelet consumption, cardiac surgery such as artificial heart valves, artificial blood vessels, vascular catheterization, and extracorporeal circulation, can cause physical damage to platelets; HAT usually occurs in the two to three days after surgery. A single-center retrospective study which enrolled nearly 14,000 non-cardiac surgery patients found that preoperative platelet transfusion did not improve the outcomes; however, these patients had lower baseline platelet levels before surgery [40]. Patients with HAT after surgery are associated with an increased risk of bleeding, transfusion risk, and mortality. Hence, there is a need for clinical based models that can identify the risk of HAT in these patients.

To date, there is a lack of accurate prediction models for predicting HAT in patients transferred to ICU after surgery. In the present report, we utilized machine learning algorithms as a novel analytic approach, since they have the property of processing big data and identifying non-linear interactions. We found that all ML models performed well, as their AUC of predicting HAT exceeded 0.7; meanwhile, the ROC analysis revealed that GB and RF had higher AUC than other models. There was no difference in AUC comparison and net benefit between GB and RF, however, each has its own advantages in sensitivity and specificity. Although both GB and RF are tree-based integrated algorithms, they are different in their method of construction and internal evaluation [41,42], thus variables importance ranking can differ among different models. Interestingly, both GB and RF models ranked the characteristic importance of platelets, procalcitonin, direct bilirubin, and prothrombin time in the top five. Although all included patients’ platelets more than 100 × 10^9^/L, the platelet count in the HAT group were much lower than non-HAT patients. Platelets have been recognized to play an important role in inflammation and immune responses, platelets release numerous inflammatory mediators that modify leukocyte and endothelial responses in the procession of inflammation [43]. This is consistent with the increase in procalcitonin in patients with HAT and the ranking of the importance of features in this study.

There are several limitations of this study. First, due to the single-center retrospective design, the ML models were derived using data only available at the time of pre-operation; therefore, the number of predictors in the models were relatively small, and models have to be extended carefully. In addition, we found that the features given by GB, RF, and XGB were concentrated and strongly related to the predicting label. Second, the models were validated in the same retrospective database. However, we used a derivation-test and 10-fold cross validation methods to reduce the overfitting of models; the models had quite discriminatory abilities (AUC) to identify patients who are more likely to develop HAT after surgery. Finally, the number of patients in the HAT group and non-HAT group were unbalanced, although we used the SMOTE method for oversampling of the HAT group; however, it was artificially synthesized new samples rather than original data.

## 5. Summary

In the current study, we constructed and validated seven supervised ML models in predicting HAT in patients transferred to ICU after surgery. We found that the AUC of ML models all exceeded 0.70, and the highest was GB (AUC = 0.834). Besides, GB and RF seemed to achieve the higher performances within these models, but there was no difference between GB and RF (0.834 vs. 0.828, *p* = 0.293). The ML models derived in the retrospective postoperative database may be a promising opportunity to predict HAT. Although external validation is necessary to improve the accuracy, this study lends substantial support to the application of ML-based prediction of the occurrence of HAT as a decision-making technology. For future researche, some novel ML algorithms such as deep learning and meta-heuristic approaches can be used to predict HAT; besides, time-series physiological data were routinely obtained in ICU and contain massive information of predicting process of disease, whether they can be used as an attempt of predicting HAT after surgery is also intriguing.

## Figures and Tables

**Table 1:** Cohort characteristics.

Variables	HAT (*n =* 1354)	Non-HAT (9015)	*p*
Age, y	57.9 ± 14.4	53.8 ± 15.2	<0.001
Male, *n* (%)	795 (58.7)	5322 (59.0)	0.815
BMI, kg/m^2^	22.98 ± 2.48	23.32 ± 2.56	0.283
Hypertension, *n* (%)	276 (20.4)	1564 (17.3)	<0.001
Diabetes, *n* (%)	154 (11.4)	785 (8.7)	<0.001
Hemoglobin, g/L	108.3 ± 23.0	115.4 ± 21.3	<0.001
Red blood cell, ×10^12^/L	3.6 ± 0.8	3.9 ± 0.7	<0.001
MCHC, g/L	327.9 ± 15.0	329.3 ± 13.7	0.001
White blood cell, ×10^9^/L	12.0 ± 5.7	12.1 ± 5.0	0.102
Platelet, ×10^9^/L	126 (111–152)	172 (137–220)	<0.001
Platelet crit	0.17 ± 0.07	0.23 ± 0.08	<0.001
Platelet distribution width	16.6 ± 4.0	15.3 ± 3.3	<0.001
Mean platelet volume, fl	12.2 ± 1.5	11.7 ± 1.4	<0.001
Hematocrit, L/L	0.33 ± 0.07	0.35 ± 0.06	<0.001
Direct bilirubin, μmol/L	8.3 (5.5–14.5)	6.1 (4.3–9.1)	<0.001
Albumin, g/L	30.7 ± 6.7	33.7 ± 6.4	<0.001
APTT, s	39.1 ± 17.3	32.4 ± 10.6	<0.001
PT, s	15.0 ± 6.3	13.0 ± 3.8	<0.001
Thrombin time, s	21.7 ± 14.0	19.4 ± 8.3	<0.001
FDP, mg/L	11.2 (6.1–21.5)	6.9 (3.5–13.4)	<0.001
Procalcitonin, ng/ml	0.6 (0.1–3.0)	0.2 (0.1–0.7)	<0.001
Interleukin-6, pg/ml	174.6 (54.7–567.4)	97.5 (29.9–282.9)	<0.001
Lactic acid, mmol/L	2.0 (1.4–3.1)	1.7 (1.3–2.7)	<0.001
Chlorine, mmol/L	108.1 ± 7.9	105.3 ± 6.4	<0.001
APACHE II	18 (13–22)	14 (9–18)	<0.001
SOFA	9.3 ± 3.4	7.2 ± 2.9	<0.001
Hospital days, d	19 (13–28)	16 (11–22)	<0.001
ICU days, d	4.8 (2.0–11.1)	2.0 (1.0–4.1)	<0.001

HAT, hospital acquired thrombocytopenia; MCHC, Mean red blood cell hemoglobin concentration; APTT, activated partial thromboplastin time; PT, Prothrombin time; FDP, fibrinogen degradation products; APACHE II, Acute Physiology and Chronic Health Evaluation; SOFA, sequential organ failure assessment.

**Table 2:** Performance of machine learning models in the test set.

ML Algorithms	AUC	95% CI	Sensitivity	PPV	Specificity	NPV
RF	0.828	0.807–0.848	0.738	34.3	0.791	95.3
GB	0.834	0.815–0.853	0.793	30.7	0.737	96.0
LR	0.798	0.773–0.822	0.780	26.8	0.686	95.5
XGB	0.801	0.780–0.823	0.845	24.6	0.619	96.4
MLP	0.804	0.782–0.826	0.720	30.4	0.757	94.8
SVM	0.704	0.679–0.729	0.685	22.1	0.649	99.1
KNN	0.708	0.679–0.736	0.710	22.9	0.647	93.8

ML, machine learning; AUC, area under the curve; CI, confidence interval; PPV, positive predict value; NPV, negative predict value; RF, random forest; GB, gradient boosting; LR, logistic regression; XGB, XGBoost; MLP, multi-layer perceptron; SVM, support vector machine; KNN, K nearest neighbor.

**Table 3:** Top five important features in ML models.

	GB	RF	XGB
1	Platelet	Platelet	Platelet
2	Procalcitonin	Direct bilirubin	Procalcitonin
3	Direct bilirubin	Procalcitonin	Interleukin-6
4	APTT	PT	APTT
5	PT	Interleukin-6	PT

ML, machine learning; GB, gradient boosting; RF, random forest; XGB, extreme gradient boosting; APTT, activated partial thromboplastin time; PT, Prothrombin time.

## Supplementary Materials

The following are available online at https://www.mdpi.com/article/10.3390/diagnostics11091614/s1, Table S1: Hyperparameters of Machine Learning models, Table S2: Baseline characteristics of derivation and test sets., Table S3: Performance of machine learning models before feature selection, Figure S1: ROC of machine learning models before feature selection.
