# Supplementary material for: Using Machine Learning Algorithms to Predict Hospital Acquired Thrombocytopenia after Operation in the Intensive Care Unit: A Retrospective Cohort Study
Source: Diagnostics (Basel). 2021 Sep 3;11(9):1614. doi: 10.3390/diagnostics11091614 (PMC8466367; doi:10.3390/diagnostics11091614)
Supplement: Supplementary file 1 [file diagnostics-11-01614-s001.zip › diagnostics-1309697-supplementary.pdf]

Supplementary Materials

Table S1. Hyperparameters of Machine Learning models.

Model	Hyperparameter	Searched value or category
RF	Number of estimators	100, 200, 300, 400, 500
	Criterion of impurity	Gini impurity, entropy
	Maximum features	3, 4, 5, 6, 7, 8, 9, 10
GB	Number of estimators	100, 200, 300, 400, 500
	Learning rate	1e-3, 1e-2, 1e-1, 1
LR	Solver	Liblinear, lbfgs, sag
	Penalty	L2, L1
XGB	eta	1e-2, 1e-1, 2e-1
	Max depth	3, 4, 5, 6, 7, 8 ,9, 10
MLP	Solver	Lbfgs, sgd, adam
	alpha	1e-5, 1e-4, 1e-3
SVM	C	1e-3, 1e-2, 1e-1, 1, 10
	Tolerance	1e-5, 1e-4, 1e-3
KNN	K	4, 5, 6, 7, 8
	weight	Uniform, distance

RF, random forest; GB, gradient boosting; LR, logistic regression; XGB, XGBoost; MLP, multi-layer perceptron; SVM, support vector machine; KNN, K-nearest neighbor.

Table S2. Baseline characteristics of derivation and test sets.

Variables	Derivation set (n=7258)	Test set (3111)	*P*
Age, y	54.5±15.2	54.2±15.1	0.225
Male, n (%)	4239 (58.4)	1878 (60.4)	0.056
BMI, kg/m^2	23.28±2.71	23.29±2.68	0.378
Hypertension, n (%)	1284 (17.7)	556 (17.9)	0.568
Diabetes, n (%)	663 (9.1)	276 (8.9)	0.361
HAT, n (%)	954 (13.1)	400 (12.9%)	0.548
Hemoglobin, g/L	114.4±21.7	114.6±21.6	0.718
Red blood cell, *10^{12}/L	3.9±0.7	3.9±0.7	0.957
MCHC, g/L	329.1±14.2	329.4±13.1	0.309
White blood cell, *10^9/L	12.0±4.9	12.1±5.5	0.376
Platelet, *10^9/L	166 (132-215)	164 (131-213)	0.319
Plateletcrit	0.23±0.08	0.23±0.08	0.344
Platelet distribution width	15.4±3.3	15.3±3.4	0.608
Mean platelet volume, fl	11.7±1.4	11.7±1.4	0.917
Hematocrit, L/L	0.35±0.06	0.35±0.06	0.898
Direct bilirubin, μmol/L	6.3 (4.4-9.6)	6.5 (4.4-9.7)	0.800
Albumin, g/L	33.3±6.5	33.3±6.6	0.704
APTT, s	33.3±12.1	33.4±11.6	0.721

PT, s	13.3±4.4	13.3±4.0	0.639
Thrombin time, s	19.7±9.5	19.6±8.8	0.414
FDP, mg/L	7.3 (3.7-14.5)	7.6 (3.8-14.6)	0.373
Procalcitonin, ng/ml	0.19 (0.06-0.88)	0.20 (0.07-0.99)	0.129
Interleukin-6, pg/ml	103.8 (32.7-308.6)	119.6 (34.2-329.4)	0.055
Lactic acid, mmol/L	1.7 (1.3-2.7)	1.8 (1.3-3.0)	0.015
Chlorine, mmol/L	105.6±6.7	105.7±6.7	0.694
APACHE II	14 (9-19)	15 (10-19)	0.451
SOFA	7.4±3.1	7.5±3.1	0.683
Hospital days, d	16 (12-23)	16 (12-23)	0.953
ICU days, d	2.2 (1.0-4.8)	2.5 (1.1-5.0)	0.226

MCHC, Mean red blood cell hemoglobin concentration; APTT, activated partial thromboplastin time; PT, Prothrombin time; FDP, Fibrin and fibrinogen degradation products; APACHE II, Acute Physiology and Chronic Health Evaluation; SOFA, sequential organ failure assessment.

Table S3. Performance of machine learning models before feature selection.

Models	AUC	95% CI	Sensitivity	PPV	Specificity	NPV
RF	0.801	0.779-0.822	0.817	25.3	0.645	96.0
GB	0.826	0.806-0.846	0.822	27.6	0.685	96.3
LR	0.792	0.768-0.817	0.741	28.6	0.730	95.0
XGB	0.787	0.764-0.811	0.686	28.6	0.749	94.2
MLP	0.795	0.773-0.817	0.709	29.1	0.746	94.6
SVM	0.700	0.675-0.725	0.739	21.1	0.594	93.9
KNN	0.702	0.674-0.730	0.736	22.1	0.620	94.1

ML, machine learning; AUC, area under the curve; CI, confidence interval; PPV, positive predict value; NPV, negative predict value; RF, random forest; GB, gradient boosting; LR, logistic regression; XGB, XGBoost; MLP, multi-layer perceptron; SVM, support vector machine; KNN, K-nearest neighbor.

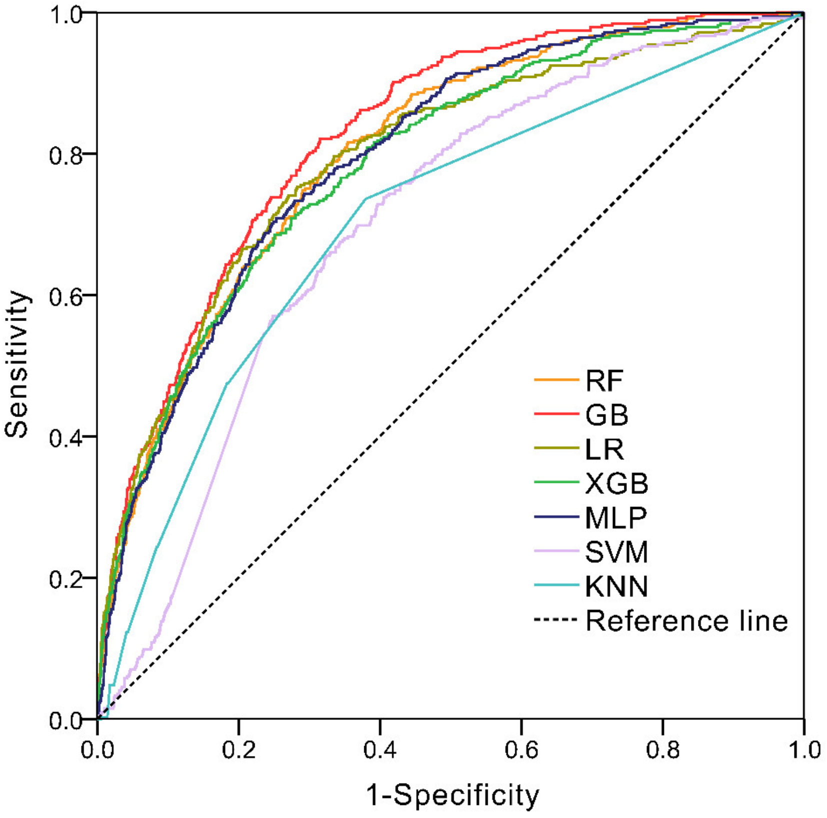

Figure S1. ROC of machine learning models before feature selection.
